# Healthcare worker long-sleeved attire contamination: a prospective observational study

**DOI:** 10.1017/ice.2025.10378

**Published:** 2026-03

**Authors:** Maria F. Sanes Guevara, Michaela C. Barry, Nathan C. Clemons, Marissa P. Griffith, Kady Waggle, Lee H. Harrison, Lora Lee Pless, Ashley M. Ayres, Graham M. Snyder

**Affiliations:** 1 Department of Medicine, UPMC Presbyterian, Pittsburgh, PA, USA; 2 Division of Infectious Diseases, Department of Medicine, Boston Medical Center, Boston, MA, USA; 3 Division of Clinical Microbiology, Department of Pathology, University of Pittsburgh School of Medicine, Pittsburgh, PA, USA; 4 Division of Infectious Diseases, Department of Medicine, University of Pittsburgh School of Medicine, Pittsburgh, PA, USA; 5 Microbial Genomics Epidemiology Laboratory, Center for Genomic Epidemiology, University of Pittsburgh, Pittsburgh, PA, USA; 6 Department of Infection Prevention and Control, UPMC Presbyterian Shadyside, Pittsburgh, PA, USA

## Abstract

**Objective:**

Estimate bacterial pathogen contamination of healthcare workers’ (HCW) long-sleeved attire.

**Design:**

Prospective observational study.

**Setting:**

Tertiary care hospital.

**Participants:**

HCWs wearing long-sleeved attire providing direct inpatient care.

**Intervention:**

Sampling of both sleeves of HCWs wearing long-sleeved attire was performed using a swab and cultured for aerobic bacterial growth classified as potential pathogens or presumptive skin commensals. Potential predictors of sleeve contamination, including participant survey responses related to attire and infection prevention practices, were analyzed using univariate analyses. Whole genome sequencing compared isolates to a genomic surveillance database of patient clinical isolates.

**Results:**

Among 280 samples, 81.1% (n = 227) demonstrated any bacterial growth and 20.7% (n = 58) grew ≥1 potential pathogen. Speciated organisms included alpha-hemolytic streptococci (n = 28), *Bacillus* sp. (n = 20), and *Pantoea*/*Mixta* sp. (n = 8), gram-negative bacilli (n = 6), and *Staphylococcus aureus* (n = 2). Univariate analysis demonstrated that sleeves sampled on non-intensive care units (*P* = .038) were significantly associated with any bacterial growth, and attire type (*P* = .002) and sleeve material (*P* = .004) were associated with growth of ≥1 potential pathogen. Fleece attire and material were more likely to be contaminated than other attire and material types. Sequenced isolates from sleeve samples were not genetically related to any patient isolates.

**Conclusions:**

HCW long sleeve contamination occurs frequently, including with potential pathogens. Changing trends in attire type may have an impact on bacterial transmissibility. While this study could not infer transmission events associated with clinically diagnosed patient infections, the potential benefit of a “bare below the elbows” attire policy warrants further investigation.

## Introduction

Studies of healthcare workers’ (HCWs) attire contamination have revealed frequent colonization by skin commensals, as well as multi-drug-resistant pathogenic bacteria.^1–3^ White coats, which are heavily represented in published attire contamination research, have been identified as frequently contaminated items of HCWs’ clothing. However, the extent to which pathogens are transmitted to patients via long-sleeved clothing, and the contribution of this contamination to hospital-acquired infections remains understudied. Some authors have demonstrated a correlation between long-sleeved attire and increased attire contamination during patient interactions, as well as genetic similarities between patient methicillin-resistant *Staphylococcus aureus* (MRSA) strains and those found on HCWs’ gowns’ sleeves and gloves.^4–7^

Based on data highlighting long-sleeved attire contamination, almost two decades ago, the United Kingdom adopted the policy “Bare Below the Elbows,” which encourages HCWs to refrain from using long-sleeved attire and hand or wrist accessories.^8^ This policy, although endorsed by expert guidance from the Society for Healthcare Epidemiology of America, is not mandatory in the United States, where attire policies are diverse and driven by hospital preferences.^2^ Some studies have explored the relationship between this policy and better hand hygiene outcomes^9–11^; however, this infection prevention strategy remains controversial since there are no studies that extensively explore the link between long-sleeved attire use and hospital-acquired infections.^12,13^

With this study, we aimed to quantify the frequency of contamination on HCWs’ long-sleeved attire. Additionally, we also aimed to characterize HCWs’ behaviors and attire characteristics associated with contamination of long-sleeved attire, and identify genetic relatedness between pathogenic bacteria isolated from these sleeve samples and isolates obtained during clinical care of patients. The goal of this quality improvement investigation was to provide guidance about attire types and practices associated with contamination and transmission risk and inform the potential impact of a “Bare Below the Elbows” attire policy.

## Methods

### Study design and setting

We conducted a prospective observational study in HCWs involved with direct patient care in critical care and non-critical care inpatient units in a 900-bed adult tertiary care hospital between April and October 2024. This study was approved by the UPMC Quality Review Committee as a quality improvement project.

### Study participants

HCWs working in inpatient medical and surgical units, including intensive care units, during first and second shift hours, were invited to participate. HCWs working in the emergency department or providing emergent care were not included. The specific days and times when sampling was conducted were chosen by a convenience sampling. The order of hospital unit participation was determined using a random number generator.

Participants included HCWs from diverse roles (nurses, nurse assistants, physicians, advanced practice providers, trainees, physical and occupational therapists, discharge planners, etc.) who were visible to the investigator during the visits to the unit, directly involved in patient care, and wearing any type of long-sleeved attire (white coat, shirt, jacket, suit, etc.) while performing patient care duties. Participation was voluntary, verbal assent was obtained, and participating HCWs were offered a five-dollar cafeteria gift card as compensation.

### Data collection

Participants were asked to complete a brief questionnaire administered by a study investigator (**Supplemental Materials,** Appendix A). No personally identifiable information was collected; the survey included a question regarding prior participation, and participants recalling previous participation or who could not recall if they previously participated were excluded. The questionnaire included the unit location, date and time, participant’s specific role, handedness, details about the long-sleeved attire to be sampled (type, material, and a visual inspection of sleeve cleanliness), and potential predictors of sleeve contamination (shift start time and the time of the last patient contact before sleeve sampling, as well as self-reported bare-below-the-elbows behaviors, adherence to standard and transmission-based precautions, and attire laundry practices). All information was recorded and stored in a secure and password-protected digital database. Participants were not informed of the culture results.

Sleeve sampling was conducted on the unit where the HCW was encountered at the time. Both HCWs and investigators performed hand hygiene and donned clean gloves before sampling. Sampling was performed using a nylon flocked swab (Eswab, Copan USA) pre-moistened with sterile normal saline solution to increase bacterial yield and transported in liquid Amies medium.^14–17^ To collect the sample, the sleeve of the participant’s dominant hand was steadied by pinching both the inner and outer surface with gloved hands; the external and most distal part of the sleeve was sampled, 5 cm from the external edge, in a circumferential manner, while the swab was twirled and rubbed back-and-forth for at least 5 seconds. The process was then repeated for the sleeve of the non-dominant hand using the same swab. Only the cuffs were sampled, based on existing literature indicating higher prevalence of contamination in this area of the sleeve.^1^ The full sampling protocol is included in the **Supplemental Materials,** Appendix B.

### Microbiological methods

Samples were transported promptly after collection to the facility’s clinical microbiology laboratory, where they were processed within 24 hours of collection. Refer to the **Supplemental Materials,** Appendix C for full laboratory methods. In brief, samples were incubated for 48 hours on both 5% sheep blood and MacConkey agar. Bacterial growth was semi-quantified as rare, light, moderate, or heavy (**Supplemental Materials,** Appendix D). All morphologically distinct gram-negative organisms that grew on MacConkey agar were speciated via matrix-assisted laser desorption/ionization (MALDI). Gram-positive organisms that grew on blood agar were classified as either non-pathogenic skin microbiota or potential pathogens based on sight identification, confirmatory Gram stain, and biochemical testing (catalase, PYR, and Staphaurex Latex Agglutination as appropriate). Hemolytic and non-hemolytic gram-positive cocci that were PYR-positive underwent MALDI for species identification. Potential pathogens were defined as organisms that were unlikely to be skin commensals, or organisms that may live on the skin but have potential to cause serious infection such as *S. aureus. Presumptive skin commensals* were not speciated.

Whole genome sequencing (WGS) was performed as previously described for all Gram-negative organisms and selected gram-positive organisms (*S. aureus*, *Enterococcus faecalis*, *Enterococcus faecium*).^18^ Genomic sequences of isolates from sleeve sampling were compared to an existing database of patient isolates from active WGS surveillance of potentially nosocomial pathogens,^18,19^ conducted from November 2021 through the present study. Isolates obtained from sleeve sampling were compared to WGS active surveillance isolates identified at least three months after completion of sleeve sampling, ensuring a window of transmission prior to sampling and following sleeve sampling.

### Statistical methods

The primary outcome was growth of any aerobic or facultative aerobic bacteria on long-sleeved attire of healthcare workers (either dominant or non-dominant sleeves), including presumptive skin commensals and/or potential pathogens. Secondary outcomes included descriptive analyses of the frequency organisms identified, the frequency of growth of potential pathogens, and predictors of sleeve contamination. For the analysis of predictors of sleeve contamination, select variables were dichotomized or categorized. Univariate analyses (Pearson’s chi-squared for categorical variables, Wilcoxon rank-sum for continuous variables) were used to analyze the relationship between potential predictors of sleeve contamination including the presence of any bacterial growth, and, separately, growth of ≥1 potential pathogenic bacteria on HCWs’ attire.

A sample size of 273 participants was estimated to provide sufficient statistical power to detect at least three transmission events; sample size assumptions included a 10% sleeve contamination rate,^2^ 11% of sleeves with growth demonstrating genetic relatedness with one or more patient isolate,^18^ alpha 0.05, beta 80%. All analyses were descriptive in nature. Stata version 12.1 was used for statistical analysis (StataCorp, College Station, Texas, USA).

## Results

Among 280 HCWs who participated in the study, registered nurses (43.2%) was the most common HCW role. The most common hospital unit types included critical care units (33.2% of observations) and medicine units (29.6%) (Table 1). Observations were made on 38 different units, with a median of 7 observations per unit (range, 1–19 observations per unit).

**Table 1.** Characteristics of study setting, and healthcare workers whose long sleeves were sampled for bacterial contamination, including the nature of care provided and attire

Variable	Study population N = 280 % (n)
Healthcare worker role	
Registered nurse	43.1 (121)
Physician trainee (resident, fellow)	18.6 (52)
Patient care technician	17.1 (48)
Medical student	6.8 (19)
Physical or occupational therapy	2.5 (7)
Respiratory therapist	2.5 (7)
Nursing student	2.1 (6)
Advanced practice provider	1.8 (5)
Discharge planner	1.8 (5)
Attending physician	1.4 (4)
Hospital unit clerk	1.1 (3)
Transport technician	0.7 (2)
Chaplain	0.4 (1)
Employment duration	
>1 year	70.0 (196)
1 month–1 year	22.5 (63)
1 week–1 month	4.6 (13)
<1 week	2.9 (8)
Unit service line	
Critical care	33.2 (93)
Medicine	29.6 (83)
Cardiothoracic	13.9 (39)
Surgical	9.3 (26)
Neurology	5.7 (16)
Trauma	5.0 (14)
Rehabilitation	1.8 (5)
Dialysis	1.4 (4)
Self-reported standard precautions adherence	
Always	66.8 (187)
Usually	32.1 (90)
Sometimes	1.1 (3)
Never	0
Self-reported transmission-based precautions adherence	
Always	57.5 (161)
Usually	35.4 (99)
Sometimes	6.8 (19)
Never	0.4 (1)
Self-reported frequency of “bare below the elbows” during care of patient on transmission-based precautions	
Always	23.2 (65)
Often	35.4 (99)
Occasionally	28.9 (81)
Never	12.5 (35)
Attire type	
Long-sleeved shirt	36.8 (103)
Suit/sport coat	28.9 (81)
Fleece	28.2 (79)
White coat	2.9 (8)
Other	1.1 (3)
Unknown	2.1 (6)
Sleeve material	
Synthetic (other than fleece)	45.4 (127)
Cotton	26.4 (74)
Fleece	25.7 (72)
Uncertain/other	0.4 (1)
Missing	2.1 (6)
Visible soiling on sleeve	
Right sleeve	8.2 (23)
Left sleeve (1 missing observation)	6.8 (19)

Participants reported “always” or “usually” adhering to standard precautions 98.9% of the time, and “always” or “usually” adhering to transmission-based precautions 92.9% of the time. Participants “always” or “often” practiced “bare below the elbows” for patient care 23.2% and 35.4% of the time, respectively (Table 1).

The time since HCW reported last laundering the long-sleeved attire was a median of 3 days (interquartile range [IQR], 1–7 days) prior to enrollment, the median (IQR) time worked during the shift at the time of sampling was 9.2 hours (7.3–10.5 hours), and the median (IQR) time since last patient contact was 0.35 hours (0.11–1.0 hours).

The type of attire varied with long-sleeved shirts (36.8%), suit or sport coat (28.9%), and fleeces (28.2%) predominating; white coats constituted fewer than 3% of long-sleeved attire. Fewer than 10% of right sleeves and left sleeves were visibly soiled (Table 1).

### Microbiological outcomes

Sleeve sampling of both sleeves was performed according to the planned procedure in all participants. Among 280 long-sleeved sampling events, 81.1% (227/280) demonstrated any bacterial growth and 20.7% (58/280) grew ≥1 potential pathogen. Among sampling events with any bacterial growth, 74.5% (169/227) grew presumptive skin commensal bacteria without potential pathogen growth, 6.6% (15/227) grew a potential pathogen without presumptive skin commensal bacteria, and 18.9% (43/227) grew both a potential pathogen and presumptive skin commensal bacteria. The specific species identified in the evaluation of potential pathogens are listed in Table 2.

**Table 2.** Microbiologic findings from sampling of healthcare workers’ long-sleeved attire for aerobic or facultative aerobic bacteria

Culture results	Frequency* N = 227	(%)
Alpha hemolytic streptococci	28	(12.3)
*Bacillus* sp.	20	(8.8)
*Pantoea/Mixta* sp.	8	(3.5)
*Acinetobacter* sp.	2	(0.88)
*Staphylococcus aureus*	2	(0.88)
*Citrobacter freundii*	1	(0.44)
*Enterococcus* sp.	1	(0.44)
*Klebsiella pneumoniae*	1	(0.44)
*Pseudomonas luteola*	1	(0.44)

*Six cultures included two potential pathogens: *Bacillus* sp. and alpha hemolytic streptococcus (2), *Bacillus* sp. and *Pantoea* sp. (2), *Bacillus* sp. and *Enterococcus* sp (1), *Acinetobacter baumannii* and *Pantoea* sp. (1).

Univariate analyses of factors potentially associated with sleeve contamination, and the outcomes of any bacterial growth and growth of ≥1 potential pathogen are presented in Table 3. Sleeves sampled on non-intensive care units were significantly more likely to demonstrate bacterial growth compared with those from intensive care units (ICU) (69.6% vs 30.4%, *P* = .038). In addition, fleece garments were more frequently associated with the growth of more than one pathogen (50%) than other attire types such as long-sleeved shirts, sport coats, or white coats (*P* = .002). Similarly, fleece material itself was more often contaminated with at least one potential pathogen (41.4%) compared with cotton or other types of synthetic fabrics (*P* = .004).

**Table 3.** Variables associated with contamination of long-sleeved healthcare worker attire with potential pathogenic bacteria. Column percentage is shown

Variable	No bacterial growth (%) N = 53	Any bacterial growth (%) N = 227	*p*-value	No bacterial growth or growth only of skin commensal (%) N = 222	Growth of ≥1 potential pathogen (%) N = 58	*p*-value
Non-intensive care unit (versus intensive care unit)	29 (54.7)	158 (69.6)	**.038**	143 (64.4)	44 (75.9)	.099
Unit service line			.13			.34
Critical care	24 (45.3)	69 (30.4)		79 (35.6)	14 (24.1)	
Medicine	8 (15.1)	75 (33.0)		65 (29.3)	18 (31.0)	
Cardiothoracic	11 (20.8)	28 (12.3)		30 (13.5)	9 (15.5)	
Surgical	3 (5.7)	23 (10.1)		18 (8.1)	8 (13.8)	
Neurology	2 (3.8)	14 (6.2)		10 (4.5)	6 (10.3)	
Trauma	3 (5.7)	11 (4.8)		13 (5.9)	1 (1.7)	
Rehabilitation	1 (1.9)	4 (1.8)		4 (1.8)	1 (1.7)	
Dialysis	1 (1.9)	3 (1.3)		3 (1.4)	1 (1.7)	
Healthcare worker role			1.00			.59
Nursing and allied health therapies	36 (67.9)	153 (67.4)		148 (66.7)	41 (70.7)	
Physician, physician trainees, and advanced practice providers	15 (28.3)	65 (28.6)		64 (28.8)	16 (27.6)	
Other professions	2 (3.8)	9 (4.0%)		10 (4.5)	1 (1.7)	
Self-reported standard precautions adherence, “always” or “usually” (compared to “sometimes” or “never”)	52 (98.1)	225 (99.1)	.52	219 (98.6)	58 (100.0)	.37
Self-reported transmission-based precautions adherence, “always” or “usually” (compared to “sometimes” or “never”)	51 (96.2)	209 (92.1)	.29	208 (93.7)	52 (89.7)	.29
Self-reported frequency of “bare below the elbows” “always” or “often” (compared to “occasionally” or “never”)	33 (62.3)	131 (57.7)	.54	132 (59.5)	32 (55.2)	.56
Attire type			.90			**.002**
Long-sleeved shirt	19 (35.8)	84 (37.0)		89 (40.1)	14 (24.1)	
Suit/sport coat	15 (28.3)	66 (29.1)		67 (30.2)	14 (24.1)	
Fleece	13 (24.5)	66 (29.1)		50 (22.5)	29 (50.0)	
White coat	2 (3.8)	6 (2.6)		7 (3.2)	1 (1.7)	
Other	0	3 (1.3)		3 (1.4)	0	
Missing	4 (7.5)	2 (0.9)		6 (2.7)	0	
Sleeve material			.94			**.004**
Synthetic (other than fleece)	24 (45.3)	103 (45.4)		108 (48.6)	19 (32.8)	
Cotton	13 (24.5)	61 (26.9)		60 (27.0)	14 (24.1)	
Fleece	12 (22.6)	60 (26.4)		48 (21.6)	24 (41.4)	
Uncertain/other	0	1 (0.4)		0	1 (1.7)	
Missing	4 (7.5)	2 (0.9)		6 (2.7)	0	
Visible soiling on right sleeve, left sleeve, or both (compared to no visible soiling on both sleeves)	3 (5.7)	27 (11.9)	.19	22 (9.9)	8 (13.8)	.39
Time since sleeved attire was laundered, days (median [IQR])*	4 (1–10)	3 (1–7)	.42	3 (1–7)	3 (1–7)	.65
Time worked that shift since sampling, hours (median [IQR])*	8.34 (7.20–10.19)	9.34 (7.33–10.55)	.23	9.11 (7.27–10.54)	9.22 (7.90–10.50)	.36
Time from last patient contact since sampling, hours (median [IQR])*	0.39 (0.11–1.61)	0.29 (0.11–1.03)	.24	0.31 (0.10–1.04)	0.35 (0.11–1.03)	.88

IQR, interquartile range.

*Unknown observations were excluded: the day long-sleeved attire was last laundered, 8 (2.9%); the time the HCW began their shift 9 (3.2%); the time the HCW last had patient contact, 10 (3.6%).

In a post-hoc ANOVA analysis of time since attire was laundered by garment type, there was not a significant difference between groups (R-squared 0.02, *P* = .24) though the mean (95% confidence interval) for HCWs wearing fleeces was longer at 15.7 (5.8–25.6) days compared to white coats at 11.5 (1.6–21.4) days and other attire types (means < 8 days).

WGS was performed on 15 isolates from 14 samples, including *S. aureus* (2), *Acinetobacter* sp. (2), *Pantoea* sp. (4), *Mixta* sp. (4), and one each *Citrobacter freundii*, *Klebsiella pneumoniae*, and *Pseudomonas luteola*. No isolates demonstrated genetic relatedness to patient isolates **(**Table 4 **)**; same species isolates from sleeve sampling were not genetically related to each other (data not shown).

**Table 4.** Number of patient isolates with existing genomic sequences compared to healthcare worker sleeve isolates

Pathogen	Number of genomic sequences
Staphylococcus aureus*	1,063
*Klebsiella pneumoniae*	397
*Citrobacter freundii*	154
*Acinetobacter baumannii*	94
*Acinetobacter nosocomialis*	13
*Pseudomonas luteola*	1

*Note. The genomic sequence database includes only methicillin-resistant strains.

## Discussion

In this prospective observational quality improvement study among diverse HCWs across multiple inpatient hospital units, we found a high rate of bacterial contamination of long-sleeved attire: 81.1% of sleeves sampled grew any bacteria, and 20.7% harbored at least one potential pathogen. Contamination was more frequently observed in non-ICU settings, and the growth of potential pathogens was significantly associated with the use of fleece garments. Visible soiling of sleeves (only 10.7% of either or both sleeves) did not predict contamination which occurred much more commonly and was not significantly correlated. Other factors, including HCW role, reported adherence to precautions, frequency of bare-below-the-elbows behaviors, laundering intervals, and duration of wear, were not significantly associated with contamination. We did not identify any genetic related pairs of HCW sleeve and patient isolates.

Our findings about attire contamination rates are consistent with previous reports documenting contamination rates from 55% to 100%,^20–30^ and pathogen recovery from 9% to 75%.^10,25,31,32^ Such heterogeneity likely reflects methodological variability, including differences in healthcare settings, HCWs’ roles, and microbiological sampling strategies.^17^ We observed a higher bacterial contamination in non-ICU settings, though this was not significant for the outcome of potential pathogens, and no differences by unit type or HCW role. The literature remains mixed in whether contamination is higher in ICU versus non-ICU settings.^20,26,27,29,32–36^ Further exploration of different variables across these settings might help clarify this difference. Across studies, however, contamination of HCWs’ attire and equipment is linked to inpatient care, particularly among staff with more frequent direct patient contact.^35,37,38^ Similarly, we did not find a significant correlation between contamination and the amount of time attire was worn or the time since last patient contact, an observation consistent with some studies,^24,27,32,33^ while others have demonstrated higher rates of bacterial contamination with longer wear durations.^39,40^

Prior research on HCWs’ attire contamination has focused largely on white coats, use of which was uncommon in our hospital. Our data suggest that other commonly worn garments, such as long-sleeved shirts under scrubs, polyester sport coats, and fleeces, also carry contamination risk. Fleece jackets were significantly more contaminated with pathogenic bacteria, in contrast to prior studies that have primarily linked bacterial growth to white coats,^3^ which as noted, represented a minority of attire sampled here. Polyester-based fabrics like fleece are known to harbor and release a greater quantity of skin bacteria compared with cotton and fabric blends.^41^ Bacteria adhere more readily to these hydrophobic materials, promoting the establishment of surface-associated biofilms that are more resistant to removal by standard home laundering.^42^ Similar to others, we found no significant relationship between laundry practices and the extent of attire contamination, likely because even freshly laundered garments become substantially contaminated within just a few hours of wear.^40,43^ Together, these data suggest potentially higher risk with this attire type.

WGS revealed no genetic relatedness between isolates recovered from HCWs’ sleeves and those obtained from patients. Previous studies demonstrating attire-to-patient transmission have largely relied on simulated conditions or involved disposable gowns.^4,5^ To our knowledge, this is the first study to use WGS to investigate whether there is a direct link between long-sleeved non-disposable attire and hospital-acquired pathogens, and the application of this technique opens a door to continue to further explore the potential transmission route. Given the predominance of non-pathogenic bacteria and inability to identify isolates genetically related to patient isolates in this study, the theoretical benefits of a bare-below-the-elbows policy may be limited. Trials of the real-world application of bare-below-the-elbows policies are warranted, particularly to incorporate other impacts such as changed hand hygiene quality and adherence.^9^ When implemented, bare-below-the-elbows recommendations should extend beyond white coats alone, to include all forms of long-sleeved garments commonly worn in hospitals.

Our study has several limitations. It was conducted at a single institution and excluded emergency department personnel, limiting generalizability. Convenience sampling and potential seasonal variation in attire use may also affect representativeness. Additionally, while the swabbing protocol was designed for thorough sampling, it was only focused on the distal sleeves, and may not capture all transmissible organisms; however, if rigorous swabbing of the most contaminated area of the attire fails to detect bacteria, it is less likely that casual patient contact would result in transmission. Our microbiological methods captured aerobic and facultative aerobic bacteria, excluding pathogens such as *Clostridioides difficile*. The WGS database, created by a prior study, does not include all potential sleeve-transmitted pathogens (e.g., methicillin-susceptible *Staphylococcus aureus*), and so we may miss potential transmission events. Furthermore, some variables (e.g., laundering intervals or perceptions of soiling) were based on self-report and may have limited accuracy. Finally, reported laundering dates, especially for long-sleeved shirts worn under scrub tops, might not indicate continuous wear over that period but rather storage of clean garments.

In conclusion, healthcare worker sleeves were contaminated with bacteria in over 80% of sampling events, with pathogenic organisms identified in 20% of cases. Higher rates of attire contamination were observed in non-critical care units, and pathogens were more commonly found on fleece jackets compared to other long-sleeved garments. No transmission events were identified using WGS. Additional studies are needed to further investigate the impact of a bare-below-the-elbows policy on hospital-acquired infections, considering all forms of long-sleeved attire and not just white coats.

## Supporting information

Sanes Guevara et al. supplementary material Sanes Guevara et al. supplementary material

## Supplementary material

The supplementary material for this article can be found at https://doi.org/10.1017/ice.2025.10378
