# Supplementary material for: Healthcare worker long-sleeved attire contamination: a prospective observational study
Source: Infect Control Hosp Epidemiol. 2025 Dec 22;47(3):277–83. doi: 10.1017/ice.2025.10378 (PMC12932925; doi:10.1017/ice.2025.10378)
Supplement: Sanes Guevara et al. supplementary material [file S0899823X25103784sup001.docx]

**Supplementary materials**

**Title:** Healthcare worker long-sleeved attire contamination: A prospective observational study

**Authors:** Maria Sanes Guevara, MD; Michaela C. Barry, MD; Nathan C. Clemons, PhD D(ABMM) MLS(ASCP); Marissa P. Griffith, BS; Kady Waggle, MS; Lee H. Harrison, MD; Lora Lee Pless, PhD; Ashley M. Ayres MBA, BS, CIC; Graham M. Snyder, MD, MS

**Table of Contents** (page)

[Appendix A: Study participant investigator-facilitated questionnaire 2](#_Toc207451857)

[Appendix B: Long sleeve sampling procedure 4](#_Toc207451858)

[Appendix C: Standard operating protocol for microbiology laboratory processing of sleeve sample specimens 6](#_Toc207451859)

# Appendix A: Study participant investigator-facilitated questionnaire

| **Question** | **Response options** |
| --- | --- |
| Study ID | [N/A – identifier] |
| Enrollment date | [date field] |
| Study team member in charge of collection | [investigators] |
| Have you previously been enrolled in this study?  *If HCW cannot recall, consider "Yes" and disqualify* | Yes  No  Cannot recall  Other |
| Unit location | [free text] |
| What is your role? | Patient care technician  Registered nurse  Respiratory therapist  PT/OT [physical therapist, occupational therapist]  MD trainee (resident, fellow)  Medical student  Nursing student  MD attending  Advanced Practice Provider  Transport technician  Hospital unit coordinator |
| How long have you worked at UPMC Presbyterian?  *If you work at Presbyterian intermittently but have not formally left your job, please consider your start date when you first worked at this hospital with the role that you currently hold.* | < 1 week  1 week - 1 month  1 month - 1 year  > 1 year |
| Date when the shift started | [date field] |
| Time when the shift started | [free text, 24-hour format] |
| How typically do you use Standard Precautions when indicated?  *Standard precautions are the minimum infection prevention practices that apply to all patients regardless of their infection status in all settings where healthcare is delivered.* | Always  Usually  Sometimes  Infrequently  Never |
| How typically do you use Transmission-based Precautions when indicated?  *Transmission-based precautions are infection prevention practices that apply to patients with suspected or known to be infected or colonized with infectious agents, including epidemiologically relevant pathogens. These include contact precautions, droplet precautions, and airborne precautions.* | Always  Usually  Sometimes  Infrequently  Never |
| Approximately what day did you last have direct patient contact?  *Date* | [date field] |
| Approximately what time on that day did you last have direct patient contact?  *Time. Please, use a 24-hour format. E.g.: 14:20. If you haven't care for a patient yet, please answer with N/A* | [free text, 24-hour format] |
| How often do you roll up your sleeves or take off your coat when providing direct patient care? | Never  Occasionally  Often  Always |
| Under what circumstances do you roll up your sleeves or take off your coat?* *Question only offered if answer to preceding question is “Occasionally”, “Often”, or “Always”* | [free text] |
| What type of long-sleeved attire are you currently wearing? | White coat  Fleece  Suit/sport coat  Long-sleeved shirt  Other |
| What type of material is your long-sleeved attire made of? | Cotton  Fleece  Synthetic (polyester and polyester combinations), Uncertain/Other |
| Approximately, how long ago did you last launder your attire? | [date field] |
| What is your dominant hand? | Right hand  Left hand  Ambidextrous |
| Was this sampling event completed? | Yes (2 sleeves/specimens)  Partially (1 sleeve/specimen)  No (0 samples submitted) |
| If only one sample was submitted, specify which side of the attire was sampled.* *Included if response to preceding question is “Partially”.* | Right  Left |
| Observer assessment of RIGHT sleeve distal to the elbow | Clean  Visible soiling |
| Observer assessment of LEFT sleeve distal to the elbow | Clean  Visible soiling |
| Did volunteer accept compensation? | Yes  No |
| Observer notes | [free text] |

*Note:*

*Responses are required for all questions except those noted with an asterisk.*

*Italicized text were included for investigator direction or response, not for healthcare worker participant/respondent.*

*Text in brackets were not included in response options but added to Appendix for clarity.*

# Appendix B: Long sleeve sampling procedure

Sample collection will be performed by study team members.

Supplies

- Tablet or Smartphone
- Disposable liner “blue pad” (1 per unit)
- E-swabs (1 per HCW)
- 5 cc 0.9% saline bullet (1 per HCW)
- 1 basin for excess saline (1 for cart)
- Gloves (2 pairs per HCW, to be worn by study team member)
- Bleach wipes (2 per HCW)
- Rolling utility cart

Storage

All the study supplies will be stored in the Infection Prevention office.

Material Transport

Materials will be brought to the predetermined unit. Hand hygiene will be performed and gloves donned by investigator responsible for sampling. The space on which initial setup will be performed will be sanitized with a bleach wipe. Materials will then be unpacked.

| **Step** | **Notes** |
| --- | --- |
| 1. Prepare space for sampling | - Sample collection will be performed at nurse’s station, team room, or rolling cart on a flat surface of at least 18” x 18“ that has been cleaned with one bleach wipe and allowed to dry - A disposable liner “blue pad” will be placed on the surface. - Study team member will perform hand hygiene, don clean gloves, and disinfect work area and tablet/smartphone used for data collection using an additional bleach wipe. - Area will be allowed to dry before preparing equipment |
| 1. Approach subjects for assent | - Available HCW will be approached and informed about the study. |
| 1. Code assignment | - Each subject will be assigned a unique identifier that includes the unit number and letter, date of data collection, and a consecutive number. |
| 1. Prepare equipment for sampling | - Set up supplies (see above) on work area - Labels will be pre-completed including the subject’s unique code and indicating the side of the sleeve being sampled. - Swabs will be pre-moistened using saline bullets. - Transport media vials will be labeled and set ready for swab placement. |
| 1. HCW positioning | - Study team member will request subjects to stand next to work area |
| 1. Hand hygiene, don clean gloves prior first sample collection | - Both study team member and subject will perform hand hygiene using sanitizer and clean don gloves |
| 1. Collect samples from the sleeves | - Sampling will be performed using a single swab.   - First, the sleeve of the dominant handwill be sampled . The swab will be applied to the external and most distal part of the sleeve, covering 5 cm from the external edge in a circumferential manner, twirling the swab while rubbing once back-and-forth covering uniformly, at least for a total of 5 seconds. The sleeve will be steadied by samplers’ hands pinching the inner and outer surface of the sleeve with gloved hands. - The same procedure will then be repeated for the sleeve of the non-dominant hand.Place the swab in the transport media and secure cap. - Tube will be labeled with unique identifier code |
| 1. Prepare samples for transport | - Place transport media tubes in biohazard bag and seal bag - Doff gloves, perform hand hygiene - Samples will be transported to Microbiology lab within 36 hours |
| 1. Complete section III of survey “Sampling” | - (see questionnaire) |
| 1. Provide HCW with $5 compensation |  |

**Specimen transport**

- Specimen collection and submission to the study laboratory will occur Mondays through Wednesdays, with submission to the laboratory by 14:00 for same day processing
- Processing of the samples will be initiated on the same day they are collected, and within 24 hours of collection.
- Specimens will be transported at ambient temperature by the study team member

# Appendix C: Standard operating protocol for microbiology laboratory processing of sleeve sample specimens

1. Semi-Quantitation

Semi-quantitate isolates in culture according to the table below

| Report As | # of Colonies  1^st^ Quadrant | # of Colonies  2^nd^ Quadrant | # of Colonies  3^rd^ Quadrant | # of Colonies  4^th^ Quadrant |
| --- | --- | --- | --- | --- |
| No Growth | 0 | 0 | 0 | 0 |
| Rare | < 10 | 0 | 0 | 0 |
| Light | > 10 | 0 | 0 | 0 |
| Moderate | > 10 | > 5 | < 5 | < 5 |
| Heavy | > 10 | > 5 | > 5 | > 5 |

1. Primary Inoculation, Isolation, & Incubation of Fomite Cultures

Perform all procedures in which infectious aerosols or splashes may be created in a biosafety cabinet using standard precautions and personal protective equipment according to established laboratory protocols.

- 1. Media: Blood agar (BAP) plate, and MacConkey agar (MAC) plate
     1. Inoculum: 1 drop on each plate near center top edge.
     2. Isolation Technique: Streak the BAP plate into 4 quadrants using 1 μL sterile plastic loops, using a fresh loop starting at the 2nd quadrant. Subsequently, with a fresh loop, streak the MAC plate into 4 quadrants using sterile plastic loops, using a fresh loop starting at the second quadrant.
     3. Incubation: 35 ± 2°C Incubator (Ambient Air & 5% CO_2_ accordingly)
     4. Incubate for 24 hours.
     5. If plate is negative for growth after 24 hours, incubate for an additional 24 hours.
     6. For plates with negative growth after 48 hours, record as negative growth and discard plates.
     7. For plates positive for growth, see section 4.4 below.

1. Work-up & Secondary Isolation for Positive Growth Fomite Cultures
   1. Gram-negative Organism Growth on MAC

All morphologically distinct gram-negative organisms that grow on MAC are to be worked up as follows:

- - 1. Semi-quantified as rare, light, moderate, or heavy
    2. Prepared to be identified via MALDI
    3. Subbed to nutrient agar (NA) slant for sequencing
    4. Organisms that cannot be isolated, identified, or subbed to NA because of scant/rare growth or poor isolation should be resubbed for secondary isolation onto BAP for another 24-48 hours.
    5. After secondary isolation, organisms are to be worked up as follows:
       1. Prepared to be Identified via MALDI
       2. Subbed to NA slant for sequencing
  1. Gram-positive Organism Growth on BAP
     1. Skin microbiota will be subject to sight ID with confirmatory gram stain and biochemical testing.
     2. Semi-quantified as rare, light, moderate, or heavy
     3. Perform Gram Stain as appropriate
     4. Perform Catalase as appropriate
     5. Perform PYR as appropriate
     6. Hemolytic and non-hemolytic Gram (+) cocci that are PYR (+) should be prepared for MALDI.
     7. Perform Staphaurex Latex Agglutination as appropriate
     8. Confirmed skin microbiota should NEITHER be prepared for MALDI NOR saved to slants for sequencing.
     9. Record results and list ID as appropriate.
  2. All other morphologically distinct gram-positive organisms (including Gram (+) PYR (+) cocci) that grow on BAP are to be worked up as follows:
     1. Semi-quantified as rare, light, moderate, or heavy
     2. Gram stained, as needed, to confirm gram-reaction
     3. Prepared to be identified via MALDI
     4. Selected organisms subbed to NA slant for sequencing.
     5. Only *S. aureus*, *E. faecalis*, and *E. faecium* are to be subbed to NA slants for sequencing.
     6. Organisms that cannot be isolated, identified, or subbed to NA because of scant/rare growth or poor isolation should be resubbed for secondary isolation onto BAP for another 24 to 48 hours.
     7. After secondary isolation, organisms are to be worked up as follows:
        1. Prepared to be identified via MALDI as appropriate
        2. Skin microbiota should not be put on the MALDI for ID.
        3. Selected organisms subbed to NA slant for sequencing.
        4. Only *S. aureus, E. faecalis,* and *E. faecium* are to be saved for sequencing*.*
        5. Skin microbiota are NOT to be saved to slants for sequencing.

1. MALDI-TOF Isolate Identification
   1. Please refer to the established MALDI-TOF ID laboratory protocol in MediaLab for isolate preparation, setup, and running the MALDI instrument.
   2. Recording of results from MALDI isolate identification output should take precedence from the Research Use Only database.
   3. Subbing of Organisms to NA
   4. Please refer to the established laboratory protocol in MediaLab for NA isolate subbing and storage for research and materials transfer.
   5. Label NA slant with Study ID number and species names

# Appendix D: Semiquantitative bacterial growth from sleeve cultures

Semiquantitative growth categories among recovered isolates

| **Growth category** | **Number of isolates** |
| --- | --- |
| One colony | 3 |
| Rare | 169 |
| Light | 102 |
| Moderate | 2 |
| Heavy | 1 |

**A total of 276 isolates were recovered across all sleeve samples.*
